# Supplementary material for: Tough Polymer Gel Electrolytes for Aluminum Secondary Batteries Based on Urea: AlCl3, Prepared by a New Solvent-Free and Scalable Procedure
Source: Polymers (Basel). 2020 Jun 12;12(6):1336. doi: 10.3390/polym12061336 (PMC7362182; doi:10.3390/polym12061336)
Supplement: Supplementary file 1 [file polymers-12-01336-s001.pdf]

SUPPLEMENTARY INFORMATION

Tough polymer gel electrolytes for aluminium secondary batteries based on urea:$AlCl_3$, prepared by a new solvent-free and scalable procedure.

Álvaro Miguel, Nuria García, Víctor Gregorio, Ana López-Cudero, Pilar Tiemblo*

Instituto de Ciencia y Tecnología de Polímeros (ICTP-CSIC), Calle Juan de la Cierva 3, 28006 Madrid,

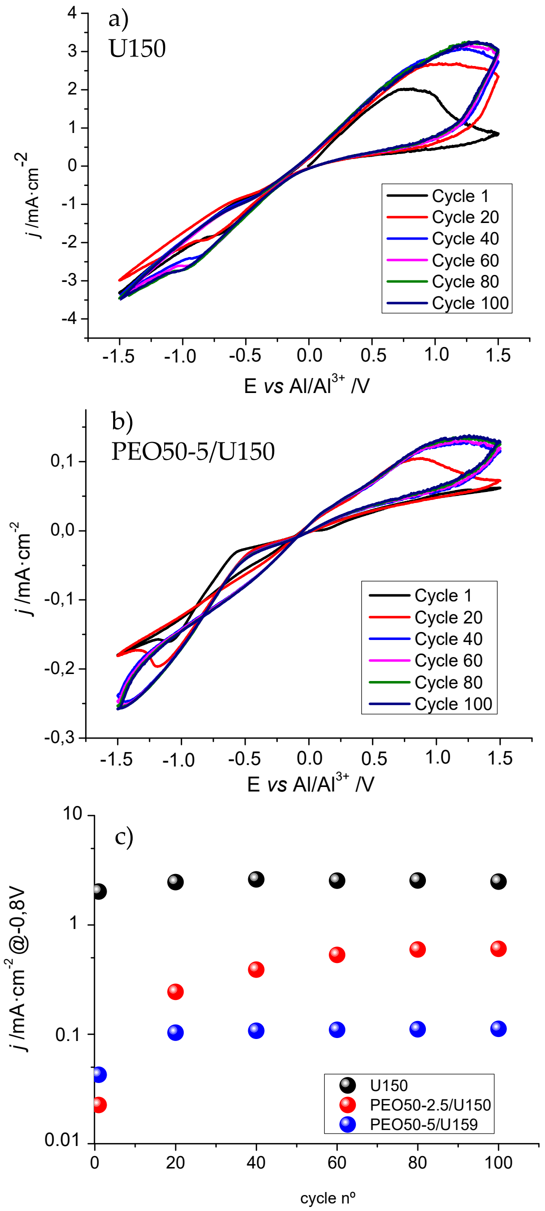

Figure S1. Selected voltagramms from cycle 1 to cycle 100 for (**a**) the less viscous pure U150 and (**b**) the most viscous gel PEO50-5/U150, showing the steady state from 40 cycles on. (**c**) Evolution on current *j* at -0.8 V for U150, PEO50-2.5/U150 and PEO50-5/from cycle 1 to 100.
